# Pediatric Early Warning Score (PEWS) in predicting prognosis of critical pediatric trauma patients: a retrospective study

**DOI:** 10.1016/j.bjane.2024.844540

**Published:** 2024-07-16

**Authors:** Abdulrahman Özel, Ulkem Kocoglu Barlas, Servet Yüce, Cansu Günerhan, Meltem Erol

**Affiliations:** a Health Sciences University Turkey, Bağcılar Training and Research Hospital, Department of Pediatrics, Pediatric Intensive Care Unit, Istanbul, Türkiye; b Istanbul Medeniyet University, Goztepe Prof Dr Süleyman Yalcin City Hospital, Department of Pediatrics, Pediatric Intensive Care Unit, Istanbul, Türkiye; c Department of Public Health, Istanbul Faculty of Medicine, Istanbul University, İstanbul, Türkiye; d Health Sciences University Turkey, Bağcılar Training and Research Hospital, Department of Pediatrics, Istanbul, Türkiye

**Keywords:** Child mortality, Critical care, Glasgow coma scale, Multiple trauma

## Abstract

**Background:**

This study aimed to compare the predictive value of Pediatric Early Warning Score (PEWS) to Pediatric Risk of Mortality-3 (PRISM-3), Pediatric Trauma Score (PTS), and Pediatric Glasgow Coma Score (pGCS) in determining clinical severity and mortality among critical pediatric trauma patients.

**Method:**

A total of 122 patients monitored due to trauma in the pediatric intensive care unit between 2020 and 2023 were included in the study. Physical examination findings, vital parameters, laboratory values, and all scoring calculations for patients during emergency room admissions and on the first day of intensive care follow-up were recorded. Comparisons were made between two groups identified as survivors and non-survivors.

**Results:**

The study included 85 (69.7%) male and 37 (30.3%) female patients, with an average age of 75 ± 59 months for all patients. Forty-one patients (33.6%) required Invasive Mechanical Ventilation (IMV) and 11 patients (9%) required inotropic therapy. Logistic regression analysis revealed a significant association between mortality and PEWS (*p* < 0.001), PRISM-3 (*p* < 0.001), PTS (*p* < 0.001), and pGCS (*p* < 0.001). Receiver operating characteristics curve analysis demonstrated that the PEWS score (cutoff > 6.5, AUC = 0.953, 95% CI 0.912–0.994) was highly predictive of mortality, showing similar performance to the PRISM-3 score (cutoff > 21, AUC = 0.999, 95% CI 0.995–1). Additionally, the PEWS score was found to be highly predictive in forecasting the need for IMV and inotropic therapy.

**Conclusion:**

The Pediatric Early Warning Score serves as a robust determinant of mortality in critical pediatric trauma patients. Simultaneously, it demonstrates strong predictability in anticipating the need for IMV and inotropic therapy.

## Introduction

Trauma is one of the primary reasons for emergency room admissions and intensive care unit stays in the pediatric age group.^1^ According to the World Health Organization (WHO) and the United Nations Children's Fund in the “World Report on Child Injury Prevention”, childhood trauma is highlighted as a significant health problem requiring urgent attention, with an annual report of approximately 950,000 children succumbing to trauma-related deaths.^2^ Scoring systems based on the initial emergency room admissions or the first signs of intensive care unit stays are employed to reduce mortality and morbidity in trauma patients. However, the evaluation of each system's feasibility and the consideration of ease of application are essential factors when using them.^3^

Currently employed scoring systems are generally complex, incorporating multiple variables and often modified versions of scoring systems designed for adults.^4^ Among these, the Pediatric Early Warning Score (PEWS) has gained increasing popularity in recent years as a scoring system for predicting early clinical deterioration in critical pediatric patients.^5^^,^^6^

The Brighton PEWS, developed in 2005 at the Brighton Children's Hospital in the UK by Monaghan et al,^5^ utilizes a scoring system based on assessing changes in the overall appearance, cardiovascular, and respiratory systems to identify clinically deteriorating patients early and initiate prompt treatment. Unlike many scoring systems, Brighton PEWS is easy and quick to apply.^7^ Its strength lies not only in predicting mortality but also in frequently being used to identify clinical severity due to its utilization of clinical findings without requiring laboratory investigations.^8^

The primary objective of this study was to compare the predictive abilities of the Brighton PEWS, Pediatric Risk of Mortality-3 (PRISM-3), Pediatric Trauma Score (PTS), and Pediatric Glasgow Coma Score (pGCS) in determining mortality in critical pediatric trauma patients monitored in the Pediatric Intensive Care Unit (PICU). The secondary objective was to compare the abilities of these scores in indicating the need for Invasive Mechanical Ventilation (IMV) and inotropic therapy in patients.

## Methods

This study was conducted retrospectively in an eight-bed PICU over a three-year period, and ethical approval for the study was obtained from the hospital's ethics committee, and the principles of the Helsinki Declaration were adhered to (Approval number: 2024/01/06/006).

Inclusion criteria for the study were: 1) Age between one month and 18 years; 2) Follow-up in the PICU due to motor vehicle accidents, falls from height, penetrating injuries, and drowning. Patients aged under one month and over 18 years, trauma patients followed in services outside the PICU, patients with underlying chronic diseases, patients intubated before arriving at the emergency department and sedated at the first encounter, and patients with data limitations were excluded from the study.

Demographic characteristics of the patients (age, sex), need for IMV, use of inotropic therapy, laboratory values (blood base parameters, glucose, potassium, urea, creatinine, white blood cell count, coagulation parameters), type of trauma, anatomical region affected by trauma (head, thorax, abdomen, and extremity), PICU and hospital length of stay, discharge-mortality status were obtained from hospital records. Brighton PEWS was calculated based on the first physical examination findings at the time of emergency room admission (Table 1); PRISM-3^9^ (Supplemental Table 1), within the first 24 hours of intensive care monitoring, with the worst laboratory values during monitoring; PTS^10^ (Supplemental Table 2) and pGCS (Supplemental Table 3) were calculated based on the vital parameters at the time of the initial emergency room admission and neurological examination findings. Patients were divided into survivors and non-survivors, and comparisons were made between the two groups.

**Table 1:** Brighton Pediatric Early Warning Score (PEWS).

	0	1	2	3
**Appearance/ Behavior**	Responsive, appropriate behaviors	Drowsy, agitated but consolable	Irritable or agitated and not consolable	Lethargic/confused **or** reduced response to painful stimuli
**Cardiovascular system**	Pink **or** Capillary refill time 1–2 seconds	Pale **or** Capillary refill time 3 seconds	Grayish **or** Cyanotic **or** Capillary refill time 4 seconds **or** Heart rate > 20 above normal	Grayish **and** mottled appearance **or** Capillary refill time ≥ 5 seconds **or** Heart rate > 30 above normal **or** Bradycardia
**Respiratory system**	Respiratory rate within normal limits, no retractions, no oxygen requirement	Respiratory rate > 10 above normal **or** Mild retractions **or** > 30% FiO_2_**or** > 3 liters/minute	Respiratory rate > 20 above normal **or** Retractions **or** > 40% FiO_2_**or** > 6 liters/minute	Respiratory rate < 5 below normal with retractions **or** Grunting **or** > 50% FiO_2_**or** > 8 liters/minute

* Scoring begins with the most severe parameter.

* An additional 2 points are added in case of persistent vomiting after surgery or every 20 minutes of nebulization (including continuous nebulization).

* Use “liters/minute” for regular nasal cannula.

* Use FiO_2_ for high-flow nasal cannula.

### Statistical analysis

IBM SPSS Statistics version 28.0 (IBM SPSS, Armonk, NY, USA) was used for statistical analyses. The data collected for the study were initially entered into the Microsoft Excel® database and then transferred to SPSS. Descriptive statistics were presented as mean ± standard deviation, median, frequency, percentage, minimum, and maximum values. The normal distribution of the data was examined using the Shapiro-Wilk and Kolmogorov-Smirnov tests. Student's *t*-test was used for the comparison of continuous variables showing a normal distribution between two groups, and one-way analysis of variance (ANOVA) was used for comparisons between more than two groups. The Mann-Whitney *U* test was employed for the analysis of continuous variables not showing a normal distribution between two groups, and the Kruskal-Wallis test was used for comparisons involving more than two groups. For the comparison of categorical variables, the Pearson chi-square test and Fisher's exact test were employed. Receiver Operating Characteristic (ROC) curves and the Area Under the Curves (AUCs) were plotted to determine specificity and sensitivity based on threshold values for the predictive powers of the scoring systems in predicting mortality, the need for IMV, and the need for inotropic therapy. All analyses conducted were two-sided, and a *p*-value < 0.05 was considered significant.

The sample size calculation was conducted using the OpenEpi open-source program. Based on this analysis, the minimum required sample size for a 95% Confidence Interval was determined to be 104 patients. Considering potential missing data, we aimed to include an additional 15%, resulting in a target sample size of 120 patients. The study was completed with 122 participants.

## Results

**Figure 1:**
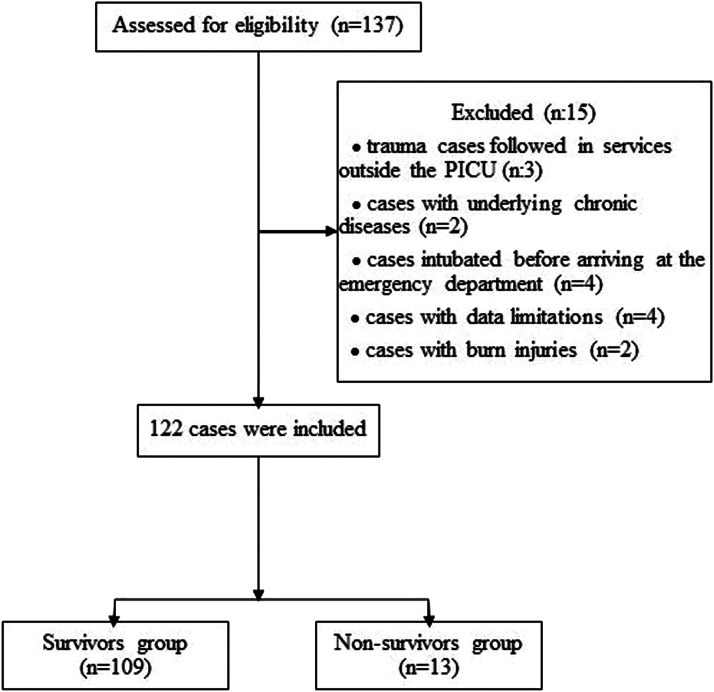
Flow chart of the selection process of study participants.

Our study included 122 pediatric trauma patients monitored in a third-level PICU between January 2020 and 2023. Figure 1 presents the selection process of study participants.

The mean age of all patients was 75 ± 59 months, 85 (69.7%) of them were male and 37 (30.3%) female. Of the patients, 55% presented to the emergency department due to falls from height, and cranial trauma was the most common type among all patients (61.5%). The demographic characteristics of all patients, the need for IMV and inotropic therapy, initial laboratory values, trauma type and region, PICU and hospital length of stay, all scoring systems, discharge-mortality status, and comparisons between survivors and non-survivors are shown in Table 2. Accordingly, there was no statistically significant relationship between the type of trauma and mortality (*p* > 0.05). The need for inotropic therapy was significantly higher in patients resulting in mortality (*p* < 0.05). PEWS and PRISM-3 scores were significantly higher in patients resulting in mortality (*p* < 0.001, *p* < 0.001, respectively), while PTS and pGCS were significantly lower (*p* < 0.001, *p* < 0.001, respectively).

**Table 2:** Evaluation of demographic characteristics, clinical findings, pediatric scores, and treatment of survivors and non-survivors.

	Survivors (n = 109)	Non-survivors (n = 13)	All patients (n = 122)	*p*
**Age, months, mean ± SD**	74.8 ± 58.25	77 ± 67.7	75 ± 59	0.891^a^
**Sex, n (%)**				0.500^b^
Male	77 (70.6%)	8 (61.5%)	85 (69.7%)	
Female	32 (29.4%)	5 (38.5%)	37 (30.3%)	
**Need for Invasive Mechanical Ventilation (IMV), n (%)**	28 (25.7%)	13 (100%)	41 (33.6%)	**<0.001**^b^^,^^c^
**Need for inotropic support, n (%)**	1 (0.9%)	10 (76.9%)	11 (9%)	**<0.001**^b^^,^^c^
**Etiology of trauma, n (%)**				
Fall from height	60 (54.1%)	8 (61.5%)	68 (55.7%)	0.891^2^
Motor vehicle accident	40 (33.9%)	4 (30.8%)	44 (36.1%)	
Penetrating injury	5 (3.7%)	1 (7.7%)	6 (4.9%)	
Drowning	4 (3.7%)	−	4 (3.3%)	
**Pathology, n (%)**				
Brain edema	12 (11.0%)	6 (46.2%)	18 (14.8%)	**<0.001**^b^^,^^c^
Intracranial hemorrhage	44 (40.4%)	11 (84.6%)	55 (45.1%)	**0.002**^b^^,^^c^
Pneumothorax	39 (35.8%)	4 (30.8%)	43 (35.2%)	0.721^b^
Pulmonary contusion	52 (47.7%)	10 (76.9%)	62 (50.8%)	**0.046**^b^^,^^c^
Hemothorax	12 (11.0%)	2 (15.4%)	14 (11.5%)	0.64^2^
Liver laceration	23 (21.1%)	1 (7.7%)	24 (19.7%)	0.25^b^
Spleen laceration	13 (11.9%)	6 (46.2%)	19 (15.6%)	**0.001**^b^^,^^c^
**Intensive Care Unit (ICU) Length of stay, days, mean ± SD**	7.51 ± 8.19	4.46 ± 4.79	7.19 ± 7.94	**0.048**^a^
**Ward length of stay, days, mean ± SD**	4.48 ± 7.97	−	4 ± 7.65	−
**pGCS, mean ± SD**	12.37 ± 3.45	4.23 ± 1.96	11.5 ± 4.17	**<0.001**^b^^,^^c^
**PEWS, mean ± SD**	2.02 ± 2.26	8.08 ± 1.71	2.66 ± 2.9	**<0.001**^b^^,^^c^
**PTS, mean ± SD**	7.17 ± 3.02	-1.08 ± 3.35	6.3 ± 3.98	**<0.001**^b^^,^^c^
**PRISM-3, mean ± SD**	3.53 ± 5.1	40.77 ± 16	7.5 ± 13.5	**<0.001**^b^^,^^c^

pGCS, Pediatric Glasgow Coma Scale; PEWS, Pediatric Early Warning Score; PTS, Pediatric Trauma Score; PRISM, Pediatric Risk of Mortality; SD, Standard Deviation; n, Number of patients; *p*, Statistical significance.

a *t*-test,

b Pearson Chi-Square test, significant p-values are highlighted in bold and indicated with the

c Symbol.

**Figure 2:**
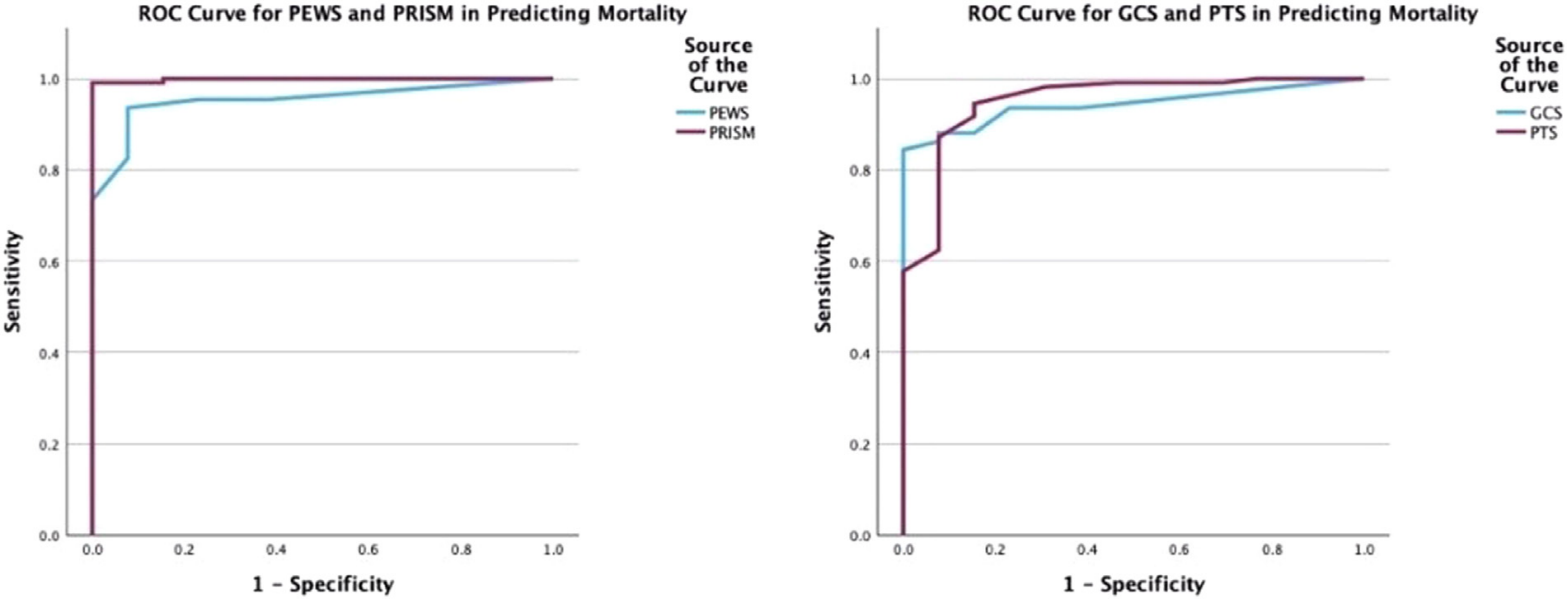
**ROC curves plotted for the Predictive Power of Scoring Systems in mortality.** (A) For the performance of pGCS in predicting mortality, the AUC was calculated as 0.943, sensitivity was 100%, and specificity was 84.4% at the cutoff of 10 (*p* < 0.001). For the performance of PTS in predicting mortality, the AUC was calculated as 0.951, sensitivity was 92.3%, and specificity was 87.2% at the cutoff of 3.5 (*p* < 0.001). (B) For the performance of PEWS in predicting mortality, the AUC was calculated as 0.953, sensitivity was 92.3%, and specificity was 93.6% at the cutoff of 6.5 (*p* < 0.001). For the performance of PRISM-3 in predicting mortality, the AUC was calculated as 0.999, sensitivity was 100%, and specificity was 99.1% at the cutoff of 21 (*p* < 0.001).

The predictive powers of the scoring systems for mortality were evaluated through ROC curve analyses in the study. Accordingly, for PEWS, with a cutoff point of 6.5, sensitivity was 92.3%, and specificity was 93.6%; for PRISM-3, with a cutoff point of 21, sensitivity was 100%, and specificity was 99.1 (Fig. 2, 2A and 2B).

**Figure 3:**
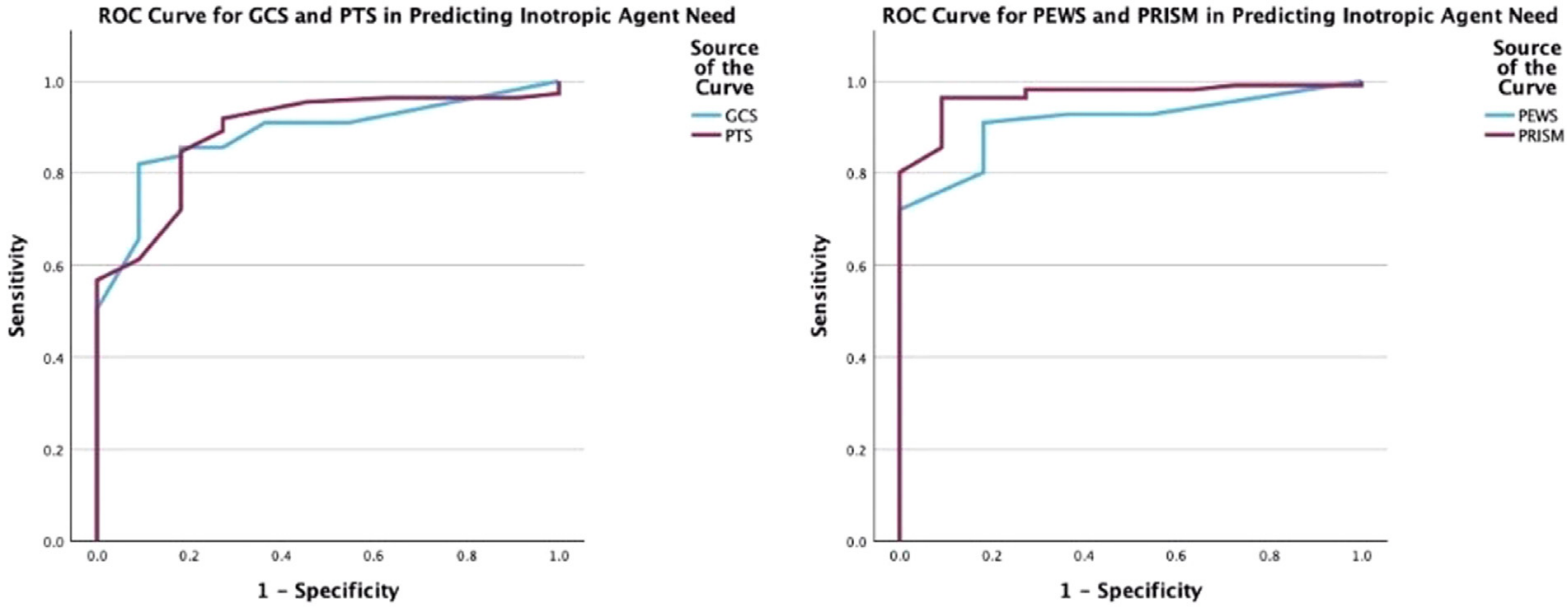
**ROC curves plotted for the Predictive Power of Scoring Systems in inotropic agent need.** (A) For the performance of pGCS in predicting inotropic agent need, the AUC for pGCS was determined to be 0.886, with a sensitivity of 90.9% and specificity of 82.0% at the cutoff value of 10 (*p* < 0.001). For predicting inotropic agent need, PTS demonstrated an AUC of 0.889, with a sensitivity of 81.8% and specificity of 84.7% at the cutoff value of 3.5 (*p* < 0.001). (B) For the performance of PEWS in predicting inotropic agent need, the AUC for PEWS was determined to be 0.912, with a sensitivity of 81.8% and specificity of 91.0% at the cutoff value of 6.5 (*p* < 0.001). For predicting inotropic agent need, PRISM-3 demonstrated an AUC of 0.968, with a sensitivity of 90.9% and specificity of 96.4% at the cutoff value of 21 (*p* < 0.001).

The predictive powers of the scoring systems in forecasting the need for inotropic therapy were evaluated through ROC curve analyses in our study. Accordingly, PEWS and PRISM-3 scores were found to have the best predictive abilities (Fig. 3, 3A and 3B).

**Figure 4:**
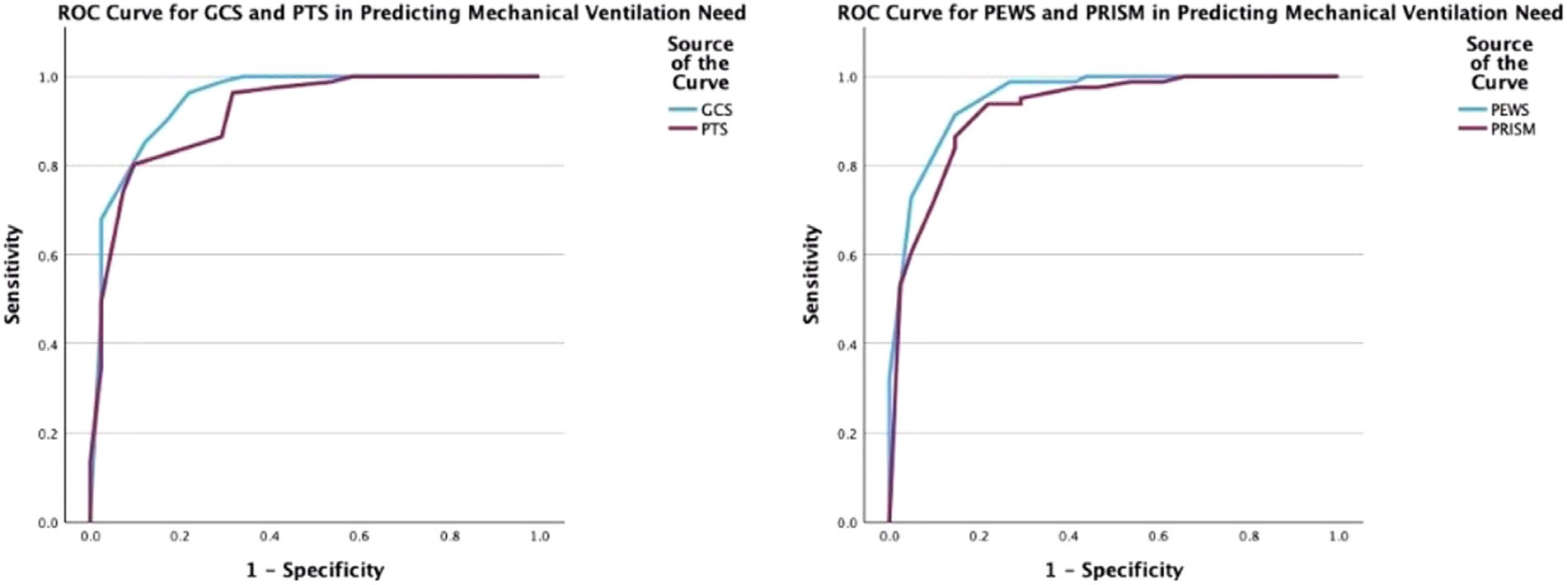
**ROC curves plotted for the Predictive Power of Scoring Systems in mechanical ventilation need.** (A) For the performance of pGCS in predicting Invasive Mechanical Ventilation (IMV) need, the AUC for pGCS was determined to be 0.947, with a sensitivity of 78.0% and specificity of 96.3% at the cutoff value of 11(*p* < 0.001). For predicting Invasive Mechanical Ventilation (IMV) need, PTS demonstrated an AUC of 0.917, with a sensitivity of 90.2% and specificity of 80.2% at the cutoff value of 6.5 (*p* < 0.001). (B) For the performance of PEWS in predicting Invasive Mechanical Ventilation (IMV) need, the AUC for PEWS was determined to be 0.951, with a sensitivity of 85.4% and specificity of 91.4% at the cutoff value of 2.5 (*p* < 0.001). For predicting Invasive Mechanical Ventilation (IMV) need, PRISM-3 demonstrated an AUC of 0.924, with a sensitivity of 78.0% and specificity of 93.8% at the cutoff value of 5.5 (*p* < 0.001).

The predictive powers of the scoring systems in forecasting the need for IMV were evaluated through ROC curve analyses. PEWS and pGCS scores were found to be the most accurate predictive systems (Fig. 4, 4A and 4B).

## Discussion

In this study, we compared the predictive abilities of PEWS, PRISM-3, PTS, and pGCS in assessing clinical severity and mortality in critical pediatric trauma patients. The results revealed that, as expected, PRISM-3 was the most sensitive score for predicting mortality, while PEWS emerged as the second most sensitive predictor, showcasing its noteworthy significance. Furthermore, PEWS demonstrated significant value in determining clinical severity.

The rapid changes in clinical findings in critically ill children have increased the importance of easily applicable and accurate scoring systems.^11^ Among these, the literature includes various studies on the PEWS, whose results have been corroborated by many researchers. For instance, Cheng et al, in their study on 4717 pediatric patients presenting to the emergency department, found the PEWS to be highly reliable in identifying patients at risk of clinical deterioration.^12^ In a study by Lillitos et al, where 273 patients were pediatric trauma patients, PEWS exhibited a specificity of 100% and sensitivity of 10% when a cutoff of three was chosen to predict severe illness.^13^ Despite our study focusing on a specific group of patients, the PEWS demonstrated excellent predictive value for mortality, akin to the PRISM-3 score (AUC: 0.953, 0.999, respectively).

The PRISM-3 score is a scoring system used in PICUs to assist clinicians in predicting mortality. Its disadvantage lies in requiring a plethora of laboratory findings and waiting for the initial 24-hour follow-up. Examining the parameters of the PEWS, it includes general appearance, cardiovascular system findings, and respiratory system findings. Post-traumatic immune system activation leads to the emergence of Systemic Inflammatory Response Syndrome (SIRS), which includes cardiovascular and respiratory system findings in its criteria.^14^ Therefore, it can be argued that PEWS is specialized in capturing early-stage SIRS patients. We believe that its superiority in predicting mortality in our study is related to this aspect. In comparison to PRISM-3, the advantage lies in being calculable at the initial presentation to the emergency department without requiring a 24-hour waiting period. This feature is particularly advantageous for critical pediatric trauma patients. It can provide insights into recognizing SIRS early, determining the treatment needs of these patients, and predicting the onset of Multiple Organ Dysfunction Syndrome (MODS). Moreover, the International Liaison Committee on Resuscitation (ILCOR) consensus, “2022 International Consensus on Cardiopulmonary Resuscitation and Emergency Cardiovascular Care Science with Treatment Recommendations”, recommends using PEWS in emergency departments to monitor hospitalized children and identify those at risk of deterioration.^15^

In our study, similar to the literature, PTS was found to be a sensitive scoring system for predicting mortality (AUC = 0.951, specificity: 87.2%, sensitivity: 92.3%). The pediatric trauma score, introduced by Tepas et al in 1987, is an easily calculable scoring system that assesses the severity of injury and indicates the risk of sudden death.^10^ Patients with a score above 8 in this scoring system have a very low probability of mortality.^16^ In the study conducted by Chabok et al^17^ on pediatric patients admitted to the intensive care unit due to trauma, the threshold for predicting mortality was ≤ 0.5 points with 100% sensitivity and 31% specificity. Similarly, in the study by Kıhtır et al on 155 pediatric patients exposed to high-energy trauma, the threshold for predicting mortality was ≤3 points with 100% sensitivity and 90% specificity.^18^

To compare the performance of scoring systems in predicting clinical severity, the need for IMV support and inotropic therapy was used. There are limited studies in the literature investigating the predictive power of the PEWS for IMV need, and these studies have mainly explored the relationship with clinical decompensation in children with respiratory diseases.^13^ In the study by Fenix et al, PEWS was found to be a valuable method in predicting clinical deterioration, and a threshold of ≥ 3 showed the best performance.^19^ In our study, in terms of predicting the need for IMV, PEWS was found to be the scoring system with the highest performance among the four scoring systems (cutoff value of 2.5, specificity: 91.4%, sensitivity: 85.4%, AUC = 0.951). We believe that, due to the majority of our patients having head trauma, the need for IMV was more related to the neurological condition than to respiratory sources. The parameters of general appearance and consciousness, included in the calculation of PEWS, provided information about the neurological condition of the patients and confirmed the need for IMV.

In predicting the need for inotropic support used to define clinical severity, PRISM-3 (AUC: 0.968, sensitivity: 90.9%, specificity: 96.4%) and PEWS (AUC: 0.912, sensitivity: 81.8%, specificity: 91%) stood out similarly regarding their performance in predicting mortality. The similar performances of scoring systems in predicting the need for inotropic support and mortality can be explained by the aggressive fluid treatment, blood product transfusion, and inotropic therapy used in the management of hypotension resulting from clinical decompensation in patients ending in mortality.

The main limitations of this study are its retrospective nature and the small number of patients. The small number of patients is attributed to being conducted in a single center and in a specific patient population. The Brighton PEWS was only evaluated based on admission findings, and repeated assessments were not performed during the intensive care follow-ups of the patients. We consider the strongest point of our study to be the first evaluation of the Brighton PEWS in critical pediatric trauma patients.

## Conclusion

In our study, the Brighton PEWS was found to be as significant as the PRISM-3 score in predicting mortality in critical pediatric trauma patients. Additionally, the Brighton PEWS was equally significant in determining the need for invasive IMV and inotropic therapy, comparable to pGCS and PRISM-3 scores, respectively. Deciding on crucial treatment options in critically ill children requires timely decision-making. The advantage of the Brighton PEWS lies in preventing the loss of time associated with waiting for laboratory results and its calculation based solely on clinical findings. To enhance these features, further studies with a larger patient population and prospective design are essential.

## Conflicts of interest

The authors declare no conflicts of interest.

## Appendix Supplementary materials

Image, application 1

## Footnotes

Supplementary material associated with this article can be found in the online version at doi:10.1016/j.bjane.2024.844540.
