# Supplementary material for: Pediatric Early Warning Score (PEWS) in predicting prognosis of critical pediatric trauma patients: a retrospective study
Source: Braz J Anesthesiol. 2024 Jul 16;74(5):844540. doi: 10.1016/j.bjane.2024.844540 (PMC11332867; doi:10.1016/j.bjane.2024.844540)
Supplement: Supplementary file 1 [file mmc1.docx]

BJORL-D-24-00089_Supplementary Material

**Supplementary Table 1** PRISM – III Score in Pediatric Patients.

|  | **Infant (1-12 months)** | **Child (1-12 years)** | | **Adolescent (> 12 years)** | **Score** |
| --- | --- | --- | --- | --- | --- |
| **Systolic Blood Pressure** | > 65 mmHg | > 75 mmHg | | > 85 mmHg | 0 |
|  | 45-65 mmHg | 55-75 mmHg | | 65-85 mmHg | 3 |
|  | < 45 mmHg | < 55 mmHg | | < 65 mmHg | 7 |
| **Heart Rate, per minute** | < 215 | < 185 | | < 145 | 0 |
|  | 215-225 | 185-205 | | 145-155 | 3 |
|  | > 225 | > 205 | | > 155 | 4 |
| **Temperature** | < 33°C | | | | 3 |
|  | 33-40°C | | | | 0 |
|  | > 40°C | | | | 3 |
| **GCS**  **Pupillary response** | < 8 | | | | 5 |
|  | Both reactive | | | | 0 |
|  | One reactive and fixed > 3 mm | | | | 7 |
|  | Both fixed > 3 mm | | | | 11 |
| **Acidosis** | pH > 7.28 | | | | 0 |
|  | pH 7-7.28, TCO_2_  5-16.9 mEq.L^-1^ | | | | 2 |
|  | pH < 7 | | | | 6 |
| **pH** | < 7.48 | | | | 0 |
|  | - 1. – 7.55 | | | | 2 |
|  | > 7.55 | | | | 3 |
| **pCO_2_** | < 50 mmHg | | | | 0 |
|  | 50-75 mmHg | | | | 1 |
|  | > 75 mmHg | | | | 3 |
| **paO_2_** | ≥ 50 mmHg | | | | 0 |
|  | 42 – 49.9 mmHg | | | | 3 |
|  | < 42 mmHg | | | | 6 |
| **Glukoz** | > 200 mg.L^-1^ | | | | 2 |
| **Potassium** | > 6.9 mEq.L^-1^ | | | | 3 |
| **Creatinine** | > 0.9 mg.dL^-1^ | | > 0.9 mg.dL^-1^ | > 1.3 mg.dL^-1^ | 2 |
| **BUN** | > 14.9 mg.dL^-1^ | | | | 3 |
| **WBC** | < 3.000 | | | | 4 |
| **Platelet** | 100.000 – 200.000 | | | | 2 |
|  | 50.000 – 99.999 | | | | 4 |
|  | < 50.000 | | | | 5 |
| **PT and PTT** | PT > 22 s and PTT > 57 s | | | | 3 |

**Supplementary Table 2** Pediatric Trauma Score (PTS).

|  | **Category** | | |
| --- | --- | --- | --- |
| **Component** | **+2** | **+1** | **-1** |
| Size | > 20 kg | 10-20 kg | < 10 kg |
| Airway | Normal | Maintainable | Unmaintainable |
| Systolic Blood Pressure | > 90 mmHg | 90 - 50 mmHg | < 50 mmHg |
| Central Nervous System | Awake | Obtunded/LOC | Coma/decerebrate |
| Open Wound | None | Minor | Major/penetrating |
| Skeletal | None | Closed fracture | Open/multiple fracture |

LOC, Level of consciousness.

**Supplementary Table 3** Modified Glasgow Coma Scale for Infants and Children.

| **Area Assessed** | **İnfant** | **Children** | **Score*** |
| --- | --- | --- | --- |
| **Eye opening** | Open spontaneously | Open spontaneously | 4 |
|  | Open in response to verbal stimuli | Open in response to verbal stimuli | 3 |
|  | Open in response to pain only | Open in response to pain only | 2 |
|  | No response | No response | 1 |
| **Verbal response** | Coos and babbles | Oriented, appropriate | 5 |
|  | Irritable cries | Confused | 4 |
|  | Cries in response to pain | Inappropriate words | 3 |
|  | Moans in response to pain | Incomprehensible words or nonspecific sounds | 2 |
|  | No response | No response | 1 |
| **Motor response^†^** | Moves spontaneously and purposefully | Obeys commands | 6 |
|  | Withdraws to touch | Localizes painful stimulus | 5 |
|  | Withdraws in response to pain | Withdraws in response to pain | 4 |
|  | Responds to pain with decorticate posturing (abnormal flexion) | Responds to pain with decorticate posturing (abnormal flexion) | 3 |
|  | Responds to pain with decerebrate posturing (abnormal extension) | Responds to pain with decerebrate posturing (abnormal extension) | 2 |
|  | No response | No response | 1 |
| * Score ≤ 12 suggests a severe head injury.  Score < 8 suggests the possible need for intubation and ventilation.  Score ≤ 6 suggests need for intracranial pressure monitoring. | | | |
| ^†^ If the patient is intubated, unconscious, or preverbal, the most important part of this scale is motor response. This section should be carefully evaluated. | | |  |
|  | | | |
